# Greenstick fractures of the proximal metaphyseal tibia: a retrospective multicenter study on the outcome after non-surgical or surgical treatment and the occurrence of posttraumatic tibia valga

**DOI:** 10.1007/s00068-022-02181-w

**Published:** 2022-12-22

**Authors:** Annabelle Weigert, Justus Lieber, Daniel Buergener, Kay Grosser, Peter Strohm, Peter P. Schmittenbecher, Joern Zwingmann, the Section of Pediatric Traumatology (SKT) of the German Association of Traumatologists (DGU)

**Affiliations:** 1 Department of Orthopedics and Trauma Surgery, Musculoskeletal University Center Munich (MUM), University Hospital, LMU Munich, Munich, Germany; 2 Department of Pediatric Surgery and Pediatric Urology, University Children’s Hospital, Tuebingen, Germany; 3 Cnopf’sche Kinderklinik Nuernberg, Nuernberg, Germany; 4 Department of Pediatric Surgery, Klinikum Kassel, Kassel, Germany; 5 Department of Traumatology and Orthopedics, Klinikum Bamberg, Bamberg, Germany; 6 Department of Pediatric Surgery, Municipal Hospital, Karlsruhe, Germany; 7 Department of Traumatology and Orthopedics, St. Elisabethen-Klinikum, Ravensburg, Germany

**Keywords:** Pediatric trauma, Lower limb, Proximal tibial fracture, Greenstick tibial fracture, Progressive valgus deformity

## Abstract

**Purpose:**

This study investigates the occurrence of (progressive) posttraumatic valgus deformity after proximal metaphyseal greenstick fractures of the tibia in young children, and whether non-surgical or surgical treatment influences the outcome.

**Methods:**

A retrospective multi-center study was conducted including surveys and X-rays of patients < 12 years of age with a fracture of the proximal tibia. In patients with greenstick fractures, the medial proximal tibia angle (MPTA; defined as the angle of the tibial axis and the joint-line of the knee) was measured at trauma, short-term follow-up (st-FU), and long-term FU (lt-FU) as defined for the 2 groups of non-surgically and surgically treated patients.

**Results:**

Of a total of 322 fractures, 91 were greenstick fractures. Of these, 74 were treated non-surgically and 17 were treated surgically. The mean MPTA at trauma of non-surgically treated patients was 91.14°, and of surgically treated patients was 95.59° (*p* = 0.020). The MPTA in non-surgically treated patients significantly increased from the timepoint of trauma to st-FU (92,0°; *p* = 0.030), and lt-FU (92,66°, *p* = 0.016). In surgically treated patients, the MTPA improved after trauma to st-FU (94.00°; *p* = 0.290), and increased again to lt-FU (96.41°; *p* = 0.618).

**Conclusion:**

Progressive valgus deformity after greenstick fractures of the proximal tibia occurred in both non-surgically and surgically treated patients. In non-surgically treated patients, this was of statistical, but not clinical significance. In surgically treated patients, progressive valgus was observed after metal removal for an unknown reason. Therefore, surgery for proximal greenstick fractures of the tibia in this age group has only limited effect and may be indicated only in selected cases. Further studies are needed to explain the responsible mechanisms.

**Level of evidence:**

III, retrospective analysis.

## Introduction

Proximal metaphyseal tibia fractures—as first described by Cozen et al. in 1953 [1]—have a low incidence with 5.6 in every 100,000 children per year [2]. Non-displaced fractures are usually treated conservatively, and in displaced fractures surgical treatment and osteosynthesis is generally performed. A frequent observation after treatment of proximal metaphyseal tibia fracture is the occurrence of progressive valgus deformity. This has been described after conservative treatment, but it has also been detected after surgical management [1–13]. Different hypotheses exist on the pathogenesis of progressive valgus deformity, such as medial invagination of the periosteum or the hamstrings, hypervascularity of medial growth plate, surgery- or manipulation-induced hypervascularity, and total or partial arrest of the lateral tibial growth plate [5, 9, 13–17]. In addition, the fracture type seems to play a decisive role in the occurrence of valgus deformity. Although ultimately rare, greenstick fractures typically occur at the proximal metaphysis of the tibia. This fracture type is characterized with a disrupted cortical bone on the convex side of the long bone, whereas the opposite cortical bone is intact [18, 19].

The aim of this study was to analyze if non-surgical or surgical treatment of greenstick fractures of the proximal metaphyseal tibia has influence on the occurrence and dynamic of a valgus deformity.

## Materials and methods

### Patients and ethical considerations

A survey was sent to 19 institutions hosting a member of the SKT to anonymously collect data of patients with a fracture of the proximal tibia treated between January 2010 and December 2019. Data on demographic characteristics, fracture type, extent of dislocation, conservative and surgical treatment, complications, and outcome were documented and stored on a computerized database. Also, X-rays of all patients were collected and analyzed by two independent clinicians using the AGFA Impax software (IMPAX AGFA, Belgium). Fractures were classified according to the AO Pediatric Comprehensive Classification of Long-Bone Fractures [19]. All data were acquired and processed according to the latest version of the World Medical Association Declaration of Helsinki—Ethical Principles for Medical Research Involving Human Subjects.

Only greenstick fractures were included in the statistical analysis. The remaining inclusion criteria were age between 0 and 11 years and the availability of X-rays (knee anterior–posterior and lateral views) at the timepoint of trauma and at least one set of X-rays during follow-up. Patients were also divided into 3 groups according to age: Group A 0–24 months, Group B 25–48 months, and Group C older than 49 months.

In all patients, the medial proximal tibia angle (MPTA) was determined on all standard anterior–posterior radiographs. To simplify analysis, we assumed the standard MPTA in a healthy lower extremity of children to be 90° even though Paley et al. reported a mean of 87.5° ± 2.5° (range 85–90) [20]. More than 90° MPTA was considered a (progressive) valgus deformity in this study.

To compare MPTA and valgus deformity between conservatively and surgically treated patients, a timeline for follow-up was defined: Short-term follow-up (st-FU) was considered the timepoint of consolidation in conservatively treated patients. In surgically treated patients, st-FU was defined immediately before or after removal of the osteosynthesis material. Long-term follow-up (lt-FU) was considered when either conservative or surgical treatment was completed and the latest X-ray had been performed.

### Statistics

Statistical analysis was performed using Student’s *t* tests (SPSS Statistics, IBM Corp., Armonk, NY, USA). Therefore, mean values of MPTA were compared within the different treatment groups, different age groups, and at the different timepoints of treatment and follow-up. All *p*-values < 0.05 were considered statistically significant.

## Results

**Fig. 1:**
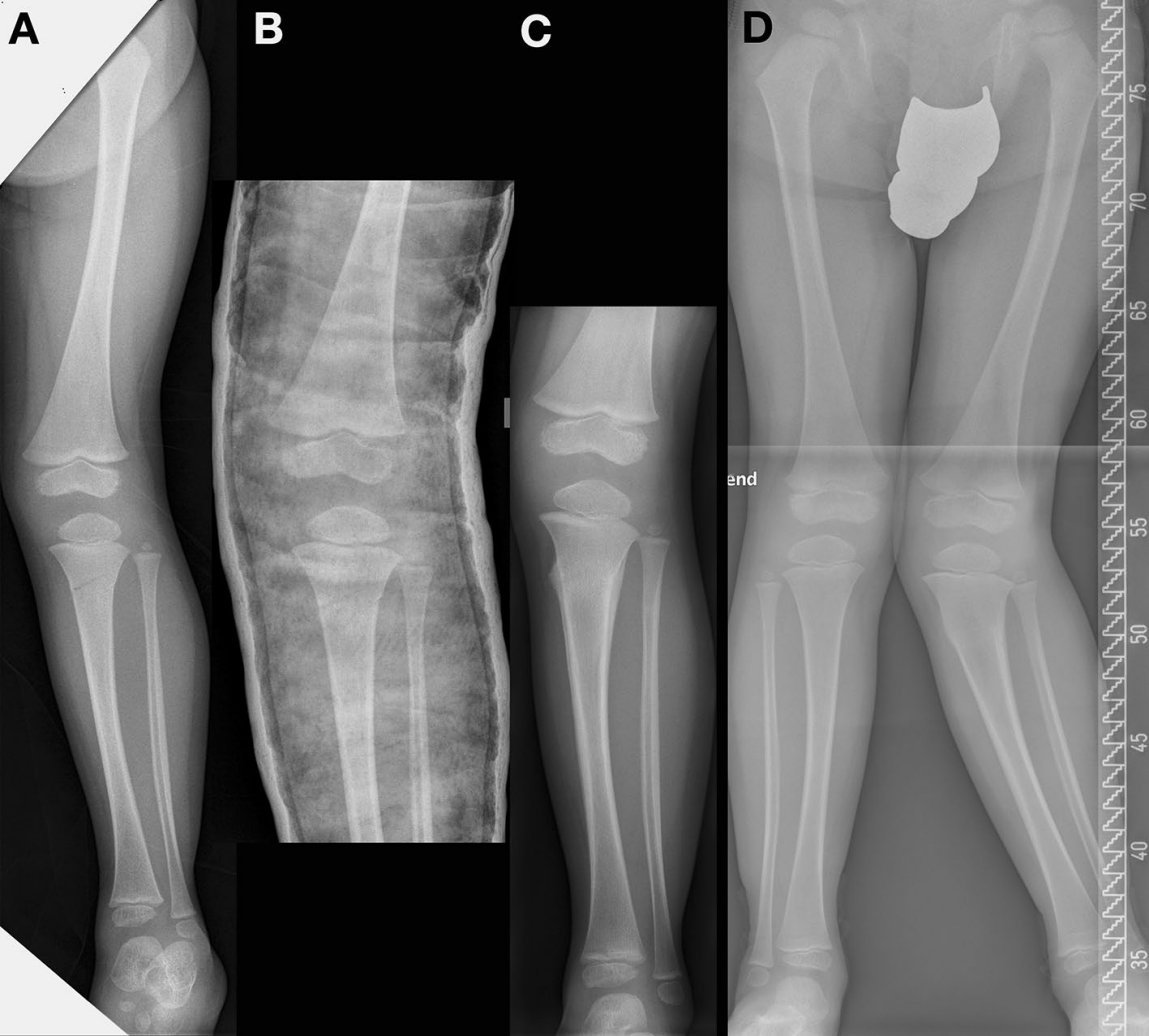
Boy (2 years and 9 months old) with a greenstick fracture of the proximal metaphyseal tibia. **A** Initial image at presentation with only a minimal gap of the fracture at the medial side of the proximal tibia. **B** Image after cast immobilization. Medial fracture gap still visible due to a lack of varus stress. **C** Consolidation after 4 weeks: development of valgus deformity detectable. **D** Progression of valgus deformity 6 months after trauma

**Fig. 2:**
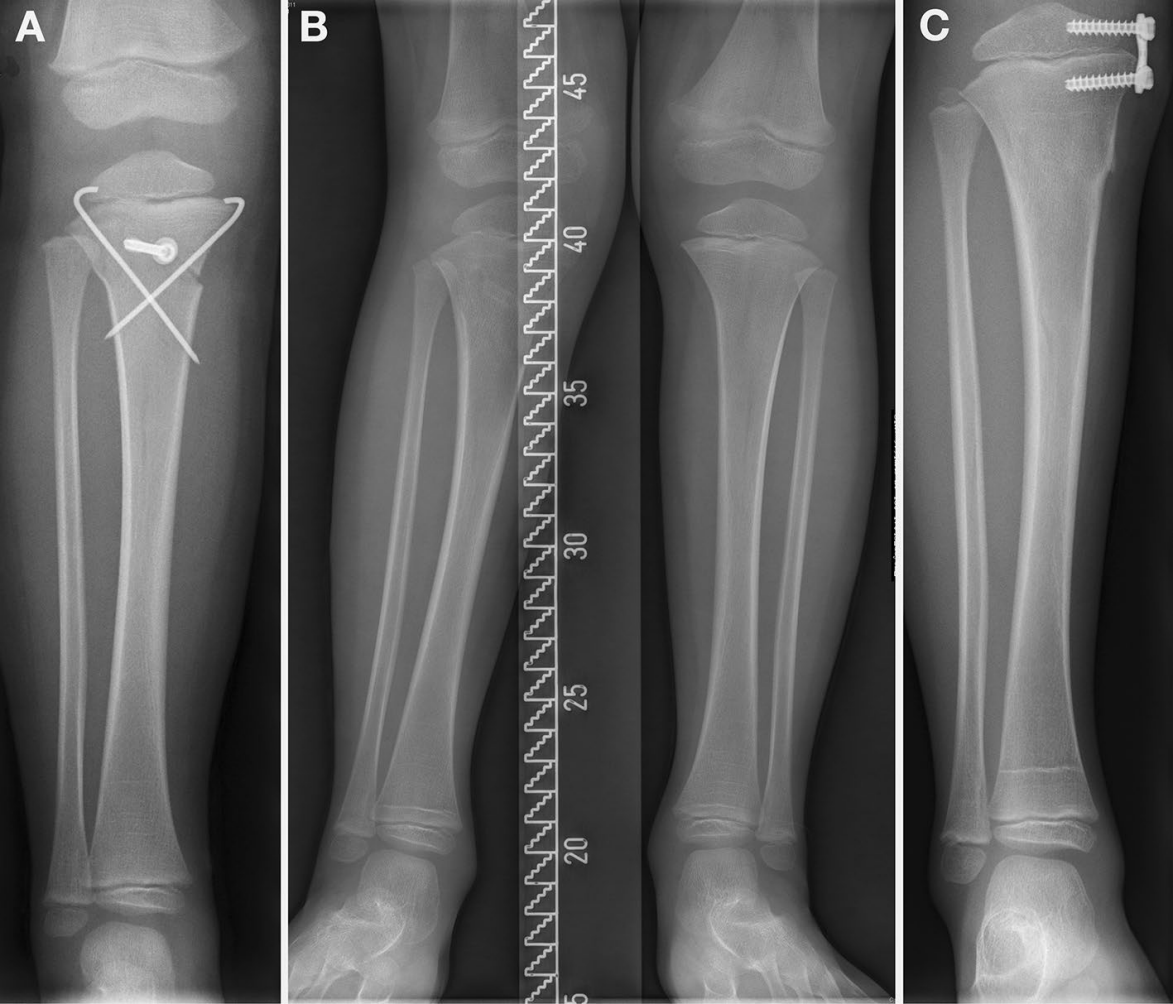
Boy (3 years and 7 months old) with a greenstick fracture of the proximal tibia. **A** Postoperative control after initial treatment with closed reduction and crossed K-wires and additional screw. **B** Status 2.5 months after removal of the K-wires and screw: development of valgus deformity. **C** Temporary epiphysiodesis 3 months later because of gait problems

A total of 322 cases were obtained through the surveys during the study period. Two-hundred complete data sets were available for evaluation [buckle fractures (*n* = 100), greenstick fractures (*n* = 91), and fractures with complete dislocation (*n* = 9)]. Demographic results of these patients and distribution of age groups A–C are shown in Table 1. Only greenstick fractures were included for further statistical analysis. Non-surgical treatment was performed in 74 patients (Fig. 1) and 17 patients received surgical treatment (Fig. 2).

**Table 1:** Incidence of fracture types and type of treatment depending on age

Group	Age		Buckle fracture	Greenstick fracture	Complete fracture
A	0–24 months	*n* =	40	17	1
		Non-surgical treatment	40	17	–
		Surgical treatment	–	–	–
B	24–48 months	*n* =	49	42	4
		Non-surgical treatment	49	37	–
		Surgical treatment	–	5	4
C	> 48 months	*n* **= **	11	32	4
		Non-surgical treatment	11	20	–
		Surgical treatment	–	12	4
Total			100	91	9

For patients with non-surgical treatment, briefly, all patients in Group A were treated conservatively using cast immobilization (Table 1). In Group B and C immobilization was realized with casts or braces, however, 8 patients also received closed reduction under analgosedation. The mean st-FU in non-surgically treated patients—meaning the timepoint of consolidation/cast removal—was 4 weeks (range 2–7). The mean lt-FU—meaning a clinical FU control of axis and movement—was 8 weeks (4–90).

Surgical treatment in 17 patients consisted of reduction under general anesthesia and osteosynthesis using either crossed K-wires (*n* = 8), ESIN (*n* = 3), external fixation (*n* = 2) or plating (*n* = 4). The mean st-FU in these patients was 10 weeks (3–52) and lt-FU 12 weeks (12–144).

**Fig. 3:**
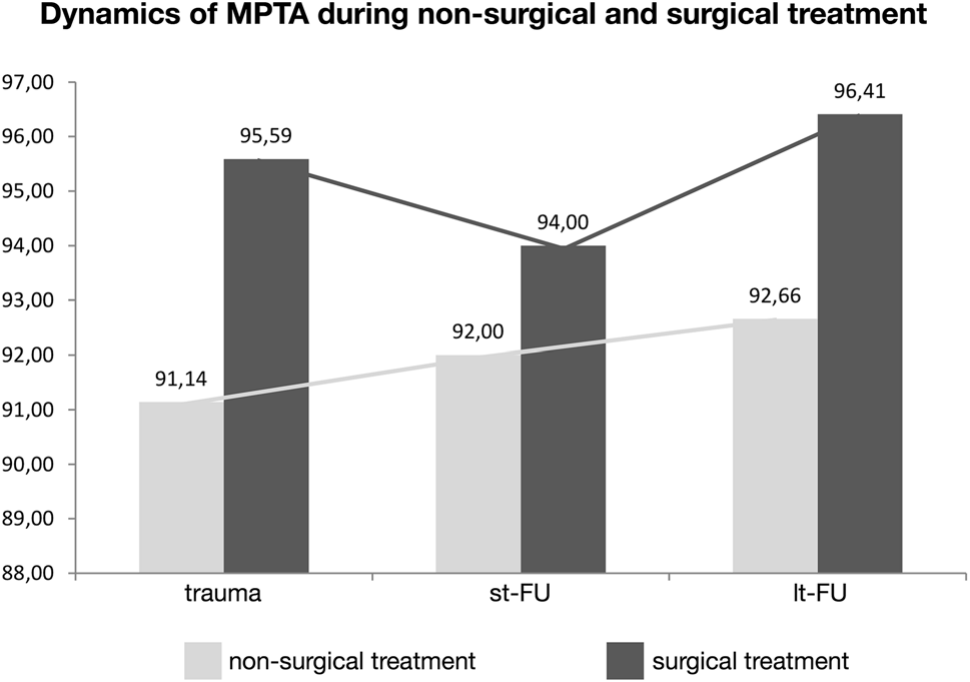
Dynamics of MPTA during non-surgical and surgical treatment. Means and standard deviation (SD) of the MPTA in greenstick fractures at trauma, st-FU, and lt-FU are shown depending on the type of treatment

Comparison of the MPTA of non-surgically and surgically treated patients at the timepoint of trauma, st-FU and lt-FU is shown in Table 2. For this, only non-surgically treated patients > 24 months of age (*n* = 57) were included due to the potential bias in results. Briefly, at the timepoint of trauma, the mean MPTA was 91.14° in non-surgically treated patients, and 95.59° in surgically treated patients (*p* = 0.020). Analysis of the progression of valgus deformity during follow-up is shown in Fig. 3. Summarizing this, MPTA in non-surgically treated patients increased from 91.14° to 92° at st-FU (*p* = 0.030), and further on to 92.66° at lt-FU (*p* = 0.016). In surgically treated patients, the MPTA at trauma (95.59°) decreased through the surgical effort to 94° (*p* = 0.290), but increased to 96.41° at lt-FU (*p* = 0.618). When comparing the mean MPTA of non-surgically and surgically treated patients at lt-FU, variances were unequal (Levene test *p* = 0.040). Therefore, statistical validation using Wilcoxon test was performed (*p* = 0.003). When analyzing MPTA depending on surgical techniques sorted in “static” fixation (external fixator, plate osteosynthesis) and “dynamic” fixation (ESIN, Kirschner-wires), no adequate statistical power could be reached, because the groups were too small.

**Table 2:** Means and standard deviation (SD) of the MPTA in Greenstick fractures depending on the type of treatment at trauma, st-FU, and lt-FU

Timepoint/type of treatment		*n*	MPTA Mean ± standard deviation (SD)	Standard mean error
Trauma	Non-surgical	57	91.14 ± 3.237	0.429
	Surgical	17	95.59 ± 6.992	1.696
st-FU	Non-surgical	57	92.00 ± 4.440	0.588
	Surgical	17	94.00 ± 5.612	1.361
lt-FU	Non-surgical	57	92.66 ± 5.199	0.695
	Surgical	17	96.41 ± 7.159	1.736

*MPTA* medial proximal tibia angle, *st-FU* short-term follow-up, *lt-FU* long-term follow-up

## Discussion

The main finding of this study is that posttraumatic progressive valgus deformity in greenstick fractures of the proximal metaphyseal tibia occurred after both surgical and non-surgical treatment. Although the cause of valgus deformity has been analyzed in several studies until now, the reason for this phenomenon is still a matter of debate. Salter [6] and Rang [21] postulate that the pre-existing valgus is the originating trigger for the later deformity. The medially located interfragmentary gap causes a delayed consolidation, which in turn results in a unilateral increase of perfusion and thus stimulates the growth plate disproportionately more, leading to progressive valgus (Fig. 1). Even a closed reduction—due to a possibly locking fibula—may not prevent this dynamic effect. Furthermore, one can hypothetically assume a biomechanical imbalance due to upward traction on the lateral side by inserting muscles and fascia lata. This could also be the explanation for the dynamic development of a valgus deformity for the patients treated conservatively in this study. To counteract this, Weber et al. [22] recommend a surgical approach with open reduction already at the timepoint of trauma. This is to ensure an anatomical reduction that fully compensates the pre-existing valgus. While performing open surgery, impacted periosteum can be removed, which may represent an obstacle to reduction. However, in this study, we could show that even after open surgery and osteo-synthetic fixation—due to some unknown mechanism—valgus deformity may occur. There is even a worsening of the valgus deformity that goes beyond the initial value at the timepoint of trauma. Overgrowth of the medial side of the physis due to surgery-induced hyperemia—analogous to the fracture hypothesis—may be possible [5]. The extent to which the initial type of osteosynthetic treatment or the subsequent metal removal plays a decisive role with regard to a temporary increase of perfusion, cannot be determined on the basis of the available data. In the present collective, open (plating) and closed (ESIN, external fixation, K-wires) osteosynthetic procedures were used, which differ in invasiveness and result in different extent of regeneration processes. In addition, static (plating, external fixation) and dynamic (ESIN, K-wires) osteosyntheses were used, which are predicted to have different effects on growth plate stimulation. However, after both static and dynamic fixation, the valgus deformity showed progression toward lt- FU. Regarding this, no statistical significance was noticed as these groups were too small for analysis.

A possible explanation for the progressive valgus in this group may be the following hypothetical fact: If valgus to some extent is left in place during surgical treatment, the same mechanisms as described for non-surgical treatment will cause progressive valgus (Fig. 2). In the present cohort, surgical treatment was initiated at a MPTA of 95.59°, but only was corrected to 94°. This is by far more than the initial value in the non-surgical group. Consequently, surgical treatment of Greenstick fractures of the proximal metaphyseal tibia presenting with valgus deviation must result in an anatomical fracture position, and realizing a stable osteo-synthesis to limit progressive valgus to its smallest extent is essential. However, this could have been only one factor among others, as one patient in the present cohort received treatment with an external fixator and still showed progressive valgus. Whether this was related to premature metal removal, impacted periosteum in the interfragmentary gap causing a delay of consolidation, or the external fixator did not provide sufficient compression, is solely speculative.

This study has some limitations which have to be addressed: One limitation of our study is the retrospective design. Patients may also have presented elsewhere and were thus lost from follow-up. In this study, we only included patients who had a documented clinical and radiological follow-up at some time point after the fracture has healed. Consequently, the total case number is reduced and thus in turn worsens statistical accuracy. Furthermore, the method of measurement for determining the MPTA is inaccurate, if one considers rotation of the limb or missing of full tibia length on some X-rays. Compatibility of the non-surgical and surgical group is also reduced, as the different treatment methods require different immobilization schedules and different indications for metal removal resulting in different timepoints for follow-ups.

The most decisive limitation of this study is the short period of follow-up. Only 16 of 91 patients exceeded the long-term follow-up with a mean of 7 months. This limitation addresses the conclusion of several authors that posttraumatic valgus deformity will spontaneously resolve on the basis of growth-related correction over time [2, 10]. However, the extent to which this happens has not been scientifically evaluated. In this series, patients with non-surgical treatment were found to have a significant MPTA-increase, but this had no clinical consequence in most cases. In contrast, 3 patients in this series received temporary epiphysiodesis to correct the deformity more quickly. Therefore, it remains unclear which amount of valgus deformity is acceptable without discomfort. In addition, the short follow-up time in this study does not take the physiological development of the leg axis from varus to valgus (affecting also the MPTA) into consideration, and therefore differentiation from processes of growth-related correction is not possible [23].

Nevertheless, the present study—although a very rare fracture—is a rather large collective compared to reports of the currently existing literature. A further attempt to query the long-term results in the same multicenter setting may answer some of the scientific questions. Analysis of the long-term course would not only provide information on the extent of spontaneous correction within the framework of physiological development of the MPTA, but also specify the surgical indication with regard to patient age and size of the interfragmentary gap. Without further long-term follow-up, surgical treatment in this series must be considered to have only limited effect and only selected patients may benefit from this type of treatment.

## Conclusion

In conclusion, progressive valgus deformity after greenstick fractures of the proximal tibia occurred in both non-surgically and surgically treated patients. In patients treated without surgery, this was of statistical, but not clinical significance. In surgically treated patients, progressive valgus was observed after metal removal for an unknown reason. Therefore, surgery for proximal greenstick fractures of the tibia in this age group has only limited effect and may be indicated only in selected cases. Further studies are needed to explain the responsible mechanisms.

## Data Availability

The datasets analyzed during the current work are available from the corresponding author upon reasonable request.
